# Enterocyte Shedding and Epithelial Lining Repair Following Ischemia of the Human Small Intestine Attenuate Inflammation

**DOI:** 10.1371/journal.pone.0007045

**Published:** 2009-09-15

**Authors:** Robert A. Matthijsen, Joep P. M. Derikx, Dian Kuipers, Ronald M. van Dam, Cornelis H. C. Dejong, Wim A. Buurman

**Affiliations:** Department of Surgery, School for Nutrition & Metabolism (NUTRIM), Maastricht University Medical Center, Maastricht, the Netherlands; Charité-Universitätsmedizin Berlin, Germany

## Abstract

**Background:**

Recently, we observed that small-intestinal ischemia and reperfusion was found to entail a rapid loss of apoptotic and necrotic cells. This study was conducted to investigate whether the observed shedding of ischemically damaged epithelial cells affects IR induced inflammation in the human small gut.

**Methods and Findings:**

Using a newly developed IR model of the human small intestine, the inflammatory response was studied on cellular, protein and mRNA level. Thirty patients were consecutively included. Part of the jejunum was subjected to 30 minutes of ischemia and variable reperfusion periods (mean reperfusion time 120 (±11) minutes). Ethical approval and informed consent were obtained. Increased plasma intestinal fatty acid binding protein (I-FABP) levels indicated loss in epithelial cell integrity in response to ischemia and reperfusion (p<0.001 vs healthy). HIF-1α gene expression doubled (p = 0.02) and C3 gene expression increased 4-fold (p = 0.01) over the course of IR. Gut barrier failure, assessed as LPS concentration in small bowel venous effluent blood, was not observed (p = 0.18). Additionally, mRNA expression of HO-1, IL-6, IL-8 did not alter. No increased expression of endothelial adhesion molecules, TNFα release, increased numbers of inflammatory cells (p = 0.71) or complement activation, assessed as activated C3 (p = 0.14), were detected in the reperfused tissue.

**Conclusions:**

In the human small intestine, thirty minutes of ischemia followed by up to 4 hours of reperfusion, does not seem to lead to an explicit inflammatory response. This may be explained by a unique mechanism of shedding of damaged enterocytes, reported for the first time by our group.

## Introduction

Recently, we demonstrated the ability of the human small intestine to rapidly restore its epithelial architecture after massive epithelial sloughing and gut barrier damage in response to a thirty minutes ischemic episode and variable reperfusion periods.[1] Interestingly, analysis of the reperfused intestinal segment demonstrated that the epithelial brush border and epithelial tight junctions were restored within the first hour of reperfusion.[1]

The presence of apoptosis or cellular damage in reperfused ischemic organs has cardinal implications in the pathogenesis of ischemia reperfusion (IR).[2], [3] Massive apoptosis and subsequent necrosis are involved in the induction of inflammation after organ ischemia. This notion is based on the observation that therapeutic strategies aimed at preventing IR induced apoptosis can ameliorate IR induced inflammation.[4]–[7] Numerous experimental studies have shown that the inflammatory response following IR mediates the development of additional reperfusion injury, further compromising organ function.[4], [8]–[12] Different strategies aimed at preventing IR induced inflammation can effectively reduce organ damage.[13]

The inflammatory response induced by IR is essentially characterized by different contributors. Early after cellular injury various cytokines, such as tumor necrosis factor-α, interleukin-1, -6, -8 or -10 and others, are expressed.[14], [15] Together with the rapid expression of adhesion molecules by activated endothelial cells, the activation, adhesion and sequestration of polymorphonuclear neutrophils (PMN) into the affected tissue is induced.[16] Infiltrating PMN contribute substantially to IR induced inflammation, by locally releasing myeloperoxidase (MPO) or generating reactive oxygen species.[10], [17] Additionally the complement system is activated, thereby triggering the formation of reactive complement split products known as anaphylatoxins, which induce additional PMN chemotaxis and cause organ damage.[18], [19] Complement activation will lead to cell damage by membrane attack complex (MAC) formation.[9], [19] Normally, under healthy conditions, these innate immune constituents protect the organism from harm by orchestrating a well mounted attack on invading microorganisms, but when faced with extensive I/R injury, sufficient means of control seem absent. In this respect, interventions aimed at preventing complement activation,[8] MAC formation,[20] expression of adhesion molecules[21] or PMN sequestration[22], [23] all prevent excessive reperfusion injury.

The presence of apoptotic and necrotic cells following ischemia reperfusion of the small intestine appears to be of critical importance in the development of an inflammatory reaction that contributes to tissue injury. From this we hypothesized that the remarkable ability of the human small intestine to effectively shed apoptotic cells, is a way of losing an important endogenous inflammatory trigger. This may serve as a unique intestinal rescue mechanism, which potentially attenuates the development of an intense IR induced inflammatory reaction. Therefore, we studied time related characteristics of IR induced inflammation of the human small intestine in a newly developed human ischemia reperfusion model of the jejunum. The results indicate that reperfused ischemic jejunum effectively discards apoptotic and damaged epithelium, restores the epithelial gut barrier and thereby prevents the development of a local inflammatory response.

## Methods

### Patients and ethics

The study was approved by the Maastricht University Medical Center ethical committee. Written consent of all patients was obtained before inclusion. Consecutively included patients were scheduled to undergo pancreatic surgery; twenty eight underwent a pylorus preserving pancreatico-duodenectomy (modified Whipple's procedure) for benign or malignant pancreatic disease and two underwent a Frey's procedure for chronic pancreatitis. During pancreatico-duodenectomy a variable length of jejunum is usually resected in continuity with the specimen. Similarly, in creating a Roux-limb for the pancreatico-jejunostomy in Frey's procedure, it is often necessary to resect a small segment of jejunum. We took advantage of this, which enabled us to study IR induced cell damage in a harmless human jejunal IR model. To that purpose, the most distal part of the jejunum to be resected with the Whipple specimen, or the most proximal part of the Roux-limb to be used as a pancreatico-jejunostomy in the Frey's procedure, was used to isolate a 6 cm segment of jejunum. The jejunum of all patients was carefully checked for any signs of underlying pathology and inflammation during operation and before the start of the IR procedure. Due to an incomplete reperfusion 2 patients were later excluded from further analysis, all having had a modified Whipple's procedure.

### Study model and blood sampling

The first part of surgery (opening of the abdominal wall, installation of a large self-retaining retractor and exploration for metastases and localization of the major anatomical structures and tumor) was according to standard procedures. From this moment the 6 cm part of jejunum, which was going to be studied, was identified and care was taken that the vasculature of the studied jejunum consisted of 1 central mesenteric arteriole and venule. This was achieved as described before.[1], [24] In brief, clamping and cutting of all collateral mesenterial vessels to the studied segment of jejunum, using Ultracision Harmonic Ace (Johnson&Johnson, cat. no. ACE23P, Amersfoort, the Netherlands). Thereafter, the segment of jejunum was further isolated by transsection at both ends with a linear cutting stapler (GIA 6038S, Covidien, Zaltbommel, the Netherlands). The isolated jejunum was then subjected to 30 minutes ischemia using 2 a-traumatic vascular clamps, which are placed over the mesentery in opposed directions to ensure complete clamping (Bulldog, Aesculap, cat. no. BH013R, Tuttlingen, Germany). The isolated ischemic jejunum was subsequently kept in the abdominal cavity to guarantee warm ischemia. After half an hour of ischemia, one third (2 cm) of the isolated ischemic jejunum was resected to study early phenomena during ischemia. Reperfusion was initiated by removal of the clamps. Adequacy of reperfusion was confirmed by the gut regaining color and restoration of peristalsis. Subsequently, the venous outflow was sampled. A further segment of isolated jejunum (2 cm) was resected similarly after 25 minutes of reperfusion to study early phenomena during reperfusion. The last part of studied jejunum was resected from 60 minutes after reperfusion onwards, to investigate late phenomena during reperfusion. At the time the last isolated reperfused segment of jejunum was obtained for the study, also 2 cm of jejunum, which had not been isolated and remained untreated during surgery, was resected using a linear cutting stapler. This tissue was used as internal control tissue: it was from the same patient and experienced similar surgical handling as the isolated jejunum, while it was not subjected to IR. Arterial blood was sampled preoperatively, before the isolated jejunum was subjected to ischemia, immediately upon reperfusion and every half hour during reperfusion until the last part of the isolated jejunum was collected at the end of the study protocol. Simultaneous with the second (i.e. before ischemia) and every next arterial blood sample, blood was drawn from the venule draining the isolated jejunal segment by direct puncture to assess concentration gradients across the isolated jejunal segment. All blood samples were directly transferred to pre-chilled EDTA vacuum tubes (BD vacutainer, Becton Dickinson Diagnostics, Aalst, Belgium) and kept on ice. At the end of the study all blood samples were centrifuged at 4000 rpm, 4°C for 15 minutes to obtain plasma. Plasma was kept on ice and immediately stored in aliquots at −80°C until analysis. The total reperfusion time was determined by the duration of the surgical procedure, with a maximum of 240 minutes. Reperfused ischemic intestine samples were obtained from every patient (n = 28). Healthy jejunum samples, serving as internal controls, were obtained from 12 patients.

### Immunohistochemistry

Paraffin-embedded sections (4 µm) were prepared from healthy and the final IR jejunum sample. These sections were stained using haematoxylin and eosin or immunostained for endothelial adhesion molecules E-selectin (CD-62-E, monoclonal antibody ENA1, kindly provided by Hycult Biotechnology (Hbt, Uden, the Netherlands)), intercellular adhesion molecule-1 (ICAM-1, monoclonal antibody HM1, Hbt) and mucosal vascular addressin cell adhesion molecule 1 (MADCAM-1, monoclonal antibody 314G8, Hbt), neutrophils by myeloperoxidase staining (polyclonal antibody, A0398, DakoCytomation, Glostrup, Denmark) or human neutrophil defensin 1–3 (monoclonal antibody, D21, Hbt) and complement component C3c (A0062, DakoCytomation). Sections were deparaffinized, rehydrated and blocked for 1 hour at room temperature using 5% BSA in phosphate buffered saline (PBS) or tris buffered saline (TBS) for HM1 and MPO. Properly diluted primary antibodies were incubated in PBS or TBS supplemented with 0.1% BSA for 1 hour at room temperature. After washing, specific antibody binding was detected using specific peroxidase conjugated secondary antibodies (Jackson Immunoresearch, West Grove, PA) or a biotinylated swine anti rabbit secondary antibody (E0432, DakoCytomation) for detecting MPO specific binding. Next, signal enhancement was achieved by peroxidase conjugated avidin biotin complexes. Staining was visualized by 3-amino-9-ethylcarbazole (AEC) followed by a hematoxylin (Sigma, St. Louis, MO) nuclear counterstaining. Microscopic analysis of several high power fields (n = 5) from different paraffin embedded tissue samples was carried out by a blinded observer.

### ELISA

Arteriovenous concentration differences of plasma Intestinal-Fatty Acid Binding Protein (I-FABP) levels were determined by Enzyme-Linked ImmunoSorbent Assay (ELISA) before and after ischemia to measure intestinal mucosal cell damage.[25] Plasma I-FABP was determined in all samples, collected at pre-fixed time points.

The I-FABP ELISA (HK406, Hbt) was performed according to the manufacturer's guidelines. Myeloperoxidase (MPO) was measured in tissue samples (healthy vs the final IR jejunum sample) that were homogenized according to the later described Western blot protocol. MPO was measured using a commercially available solid-phase ELISA kit based on the sandwich principle (Hbt) in aliquots containing equal amounts (10 µg) of total protein. The assay was performed in accordance with the manufacturer's guidelines.

Arteriovenous concentration differences in plasma tumor necrosis factor-α (TNFα) levels were determined by ELISA before and after ischemia as well as during reperfusion. The TNFα ELISA was performed as described before.[26] Arteriovenous concentration differences in plasma lipopolysacharide (LPS) levels were determined using a Limulus Amebocyte Lysate (LAL) assay before and after ischemia as well as during reperfusion. The LAL assay (HIT 302, Hbt) was performed in accordance with the manufacturer's guidelines. Plasma LPS was determined in all samples, collected at pre-fixed time points.

### Western Blot

Western blot analyses for human C3 deposition on reperfused ischemic tissue samples and healthy controls was performed as described before, with minor modifications.[4] Jejunal samples were homogenized in lysis buffer (200 mM NaCl, 10 mM Tris base, 5 mM EDTA, 10% Glycerin, 1 mM PMSF, 0.1 U/ml Aprotinin and 1 µg/ml Leupeptin). Tissue homogenates were centrifuged. Protein concentrations of the different lysates were determined using Bradford analyses. Aliquots containing equal amounts (10 µg) of total protein were heated and reduced, transferred to an 8% SDS-polyacrylamide gel and blotted on an Immobilon-P Polyvinylidene Difluoride (PVDF) membrane (Millipore, Bedford, MA). Blocking was performed in phosphate buffered saline (PBS) containing 5% bovine serum albumin and 0.1% Tween-20 (Sigma). C3 was detected in PBS 0.1% BSA 0.1% Tween-20 after overnight incubation of the properly diluted rabbit anti human C3c (A0062, DakoCytomation, Glostrup, Denmark). Binding of the primary antibody was detected using a biotinylated secondary antibody to rabbit IgG (E0353, Dako) and streptavidin conjugated peroxidase. Positive bands were visualized using chemiluminescence (Supersignal (pico:femto 9∶1), Pierce, Rockford, IL).

### Real-Time Quantitative - PCR analysis

Total RNA was extracted from healthy and all IR damaged jejunum samples using a SV Total RNA Isolation System according to manufacturer's guidelines (Promega, Madison, WI). Total cDNA was synthesized with oligo (dT) primers and Molony murine leukemia virus reverse transcriptase. Specific primers (Sigma) for amplification of hemoxygenase-1, hypoxia inducible factor 1-α (HIF-1α), interleukin-6 and -8, TNFα and complement component C3 specific cDNA were designed (Table 1) using the Primer Express software package (Applied Biosystems, Foster City, CA) and tested for amplification of contaminating genomic DNA. To minimize the risk of genomic amplification the primers were positioned on different exons. Primer concentrations were optimalized and dilution curves were made from human liver cDNA standard pool to ensure an exponential Taqman amplification for each primer set. After normalization to endogenous reference genes, RPLP0 and cyclophylin-A, the level of expression of different proteins in healthy or IR damaged samples was determined by the comparative ΔΔC_T_ method of relative quantification. Primer specifications are listed in Table 1.

**Table 1:** Sequence of primers for Q-PCR analysis.

Proteins	Primers
	Forward	Reversed
HIF-1α	3-TGAACATAAAGTCTGCAACATGGA-5	5-TGAGGTTGGTTACTGTTGGTATCATATA-3
HO-1	3-CTTCTTCACCTTCCCCAACA-5	5-GCTCTGGTCCTTGGTGTCAT-3
TNFα	3-TCAATCGGCCCGACTATCTC-5	5-CAGGGCAATGATCCCAAAGT-3
IL-6	3-TCCAGGAGCCCAGCTATGAA-5	5-GAGCAGCCCCAGGGAGAA-3
IL-8	3-CTGGGCGTGGCTCTCTTG-5	5-TTAGCACTCCTTGGCAAAACTC-3
C3	3-CCCTCATCATCTACCTGGACAAG-5	5-GCTGGATAAGCTCTACATTAAAGTATTGG-3
RPLP-0	3-GCAATGTTGCCAGTGTCTG-5	5-GCCTTGACCTTTTCAGCAA-3
Cycophilin A	3-CTCGAATAAGTTTGACTTGTGTTT-5	5-CTAGGCATGGGAGGGAACA-3

### Statistical analysis

Data are expressed as means ± SEM and were analyzed by either paired or unpaired two-tailed Student's t-test depending on sample collection. Bonferroni's multiple comparison test was used (after significant repeated measures ANOVA) to compare I-FABP or LPS arteriovenous concentration differences in time. Data were analyzed using Prism 4.01 for Windows (Graphpad Software, San Diego, CA). A p-value≤0.05 was considered statistically significant.

## Results

### Patient outcome and reperfusion period

**Figure 1:**
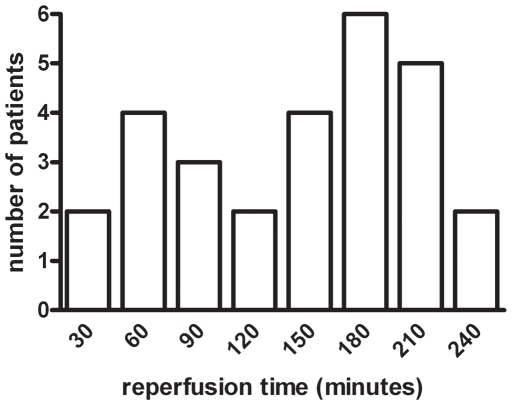
Reperfusion times. Distribution of maximal reperfusion times of the isolated jejunum, following 30 minutes of ischemia (n = 28).

The mean (± SEM) age of included patients (n = 28) was 65 (±2) years and 64% were male. Isolated jejunum samples were subjected to 30 minutes of ischemia followed by a mean total reperfusion time of 120 (±11) minutes (Fig. 1).

### Intestinal epithelial damage

**Figure 2:**
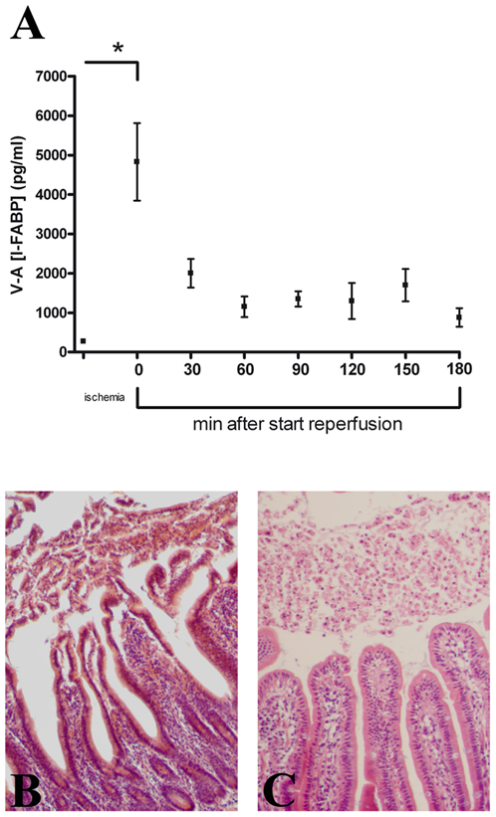
Epithelial cell damage. A) Arteriovenous concentration differences of I-FABP (mesenteric venule minus radial artery) across the isolated ischemic jejunal segment show rapid release of I-FABP into the circulation at reperfusion (early R vs healthy * p<0.001). B) Shedding of damaged epithelial cells into the intestinal lumen during the first thirty minutes of reperfusion. C) Rapid repair of the intestinal epithelial lining, observed following 1 hour of reperfusion.

Epithelial cell integrity was lost in response to 30 minutes of ischemia followed by various periods of reperfusion. This was illustrated by increased plasma I-FABP levels in the venous outflow of the reperfused jejunum, when compared to arterial circulating levels and I-FABP levels prior to the IR period (* p<0.001. Fig. 2A). As described by Derikx et al. rapid sloughing of intestinal epithelial cells, during the first thirty minutes of reperfusion, was observed. Shedding of epithelial cells was followed by a full and rapid repair of the damaged intestinal epithelial barrier within the first hour of reperfusion (Fig. 2B–C).[1]

### Plasma LPS levels following IR

**Figure 3:**
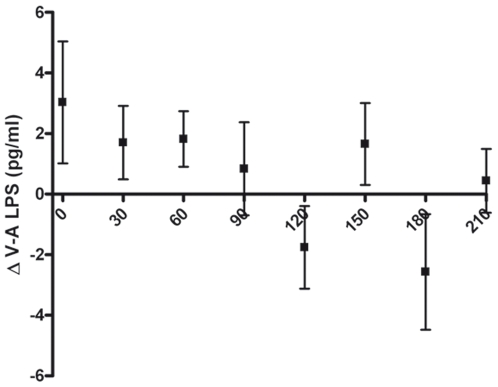
Tissue response to IR. Illustrative of rapid gut barrier repair, no translocation of LPS could be detected during the IR procedure. Translocation of LPS was calculated from arteriovenous differences in LPS plasma levels across the isolated bowel segment during reperfusion (Δ (V–A). p = 0.18).

Disruption of the gut barrier may lead to translocation of microbiota and pro-inflammatory microbial products, such as endotoxin associated with gram negative bacteria (lipopolysacharide (LPS)). Therefore we assessed the translocation of LPS following the rapid and temporary disruption of the epithelial lining in response to IR observed in our model.[1] LPS translocation from the intestinal lumen was assessed by measuring arterio-venous (AV) differences in LPS concentration across the jejunal segment, using the LAL-assay. Surprisingly, no translocation of LPS (p = 0.18, n = 28) was detected at any time point during reperfusion (Fig. 3). The temporary loss of gut wall integrity observed during reperfusion, which is induced by massive shedding of apoptotic epithelial cells, does not lead to the translocation of LPS.

### Stress response

In order to demonstrate a physiological response to anoxia and to reperfusion stress, the mRNA expression profiles of hypoxia inducible factor-1α (HIF-1α) and heme oxygenase-1 (HO-1) were determined in healthy and IR damaged whole jejunum samples (n = 12). The HIF-1α expression, which regulates both physiologic and pathophysiologic responses to ischemia, increased during reperfusion (Table 2). In contrast, local mRNA expression of HO-1 was unaltered by IR (Table 2).

**Table 2:** mRNA expression profiles in response to IR, calculated by comparative ΔΔC_T_ method of relative quantification.

Gene	Healthy	IR	Fold increase (95%-CI)	P - value
HIF-1α	1.00	1.91	1.91 (1.15–2.67)	0.02*
HO-1	1.00	1.58	1.58 (0.22–2.94)	0.34
TNFα	1.00	1.18	1.18 (0.56–1.80)	0.54
IL-6	1.00	12.31	12.31 (−5.29−29.91)	0.18
IL-8	1.00	15.86	15.86 (0.59–31.13)	0.06
C3	1.00	4.09	4.09 (1.74–6.43)	0.01*

* p-value≤0.05 was considered statistically significant (n = 12).

### Cytokine expression and endothelial activation

In order to assess the inflammatory reaction over the course of IR, serial venous blood samples from the reperfused jejunum were analyzed for different inflammatory markers. Additionally, the cytokine response in reperfused whole jejunum tissue samples was studied using Q-PCR. Endothelial cell activation was assessed by immunohistochemistry on paraffin embedded whole jejunum tissue samples before and after IR. No increase in TNFα protein levels was detected in venous plasma samples, collected from the isolated jejunum during the procedure; e.g. prior to ischemia, directly after ischemia and every 30 minutes during reperfusion. Compared to the TNFα levels in arterial circulation, AV-differences in TNFα levels revealed no release of TNFα from the gut (data not shown). In line, no increase in TNFα mRNA was detected in IR damaged jejunum samples, when compared to healthy jejunum controls from the same patient (Table 2).

mRNA expression of cytokines IL-6 (Table 2) and IL-8 (Table 2) in response to IR differed widely and failed to show an increase (n = 12). The reperfusion length did not show any relation with maximal IL-6 or IL-8 mRNA levels (data not shown).

**Figure 4:**
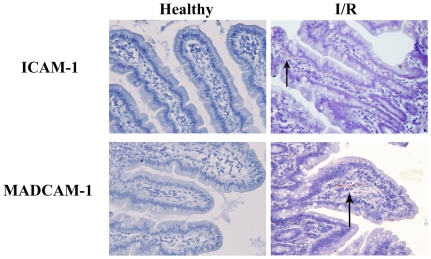
Endothelial activation. Compared to healthy control jejunum tissue, reperfused jejunum samples in response to 30 minutes of ischemia show an only moderate increase in ICAM-1 (upper panel) and MADCAM-1 (lower panel) expression demonstrated by terracotta red staining of the AEC (indicated by arrows). Original magnification 200x.

Classic in the development of extensive IR damage is the early expression of various adhesion molecules by activated endothelial cells. Presence of E-selectin, ICAM-1 and MADCAM-1 were analyzed using immunohistochemistry. Only 3 patient samples demonstrated sporadic and minimal expression of E-selectin (data not shown), ICAM-1 and MADCAM-1 (Fig. 4) following maximal reperfusion.

### Neutrophil infiltration and release of myeloperoxidase

**Figure 5:**
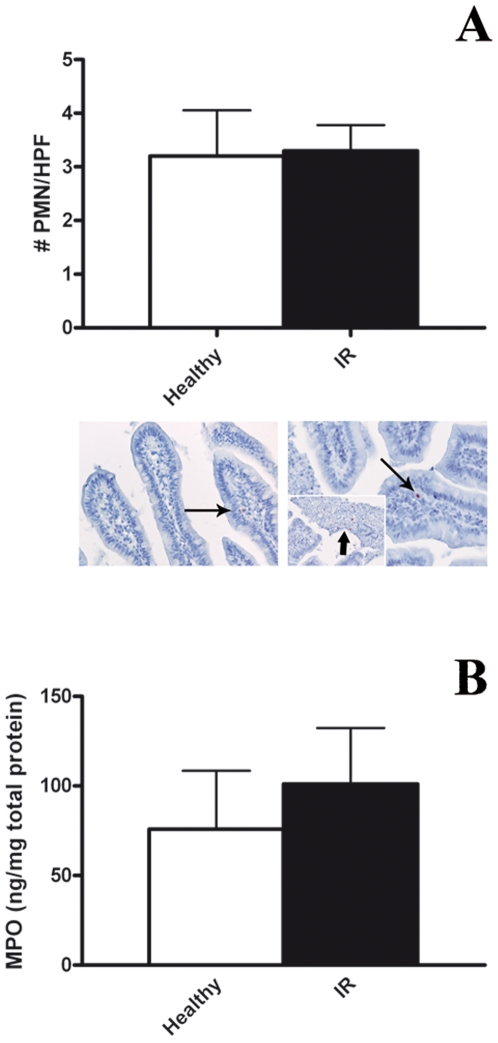
Neutrophil recruitment. A) No increase of PMN was observed over the course of reperfusion in response to 30 minutes of ischemia. Detected by specific HNP1-3 staining (AEC, indicated by arrows) the number of PMN in reperfused jejunum did not increase in comparison to healthy tissue (p = 0.90). Original magnification 200x. Clearly the PMN concentrated around the cellular debris collecting in the safe intestinal lumen (right insert in Fig. 4A indicated by arrow. Original magnification 200x). B) Tissue MPO, assessed by ELISA, did not increase substantially over the course of IR (p = 0.71).

Critical in the induction of reperfusion injury is the influx of neutrophils as well as the release of their reactive constituents. The presence of neutrophils was assessed using two different and widely used neutrophil markers, human neutrophil defensin 1–3 (HNP1-3) and myeloperoxidase (MPO), both stored in abundance in azurophilic granules of neutrophils. Analysis of immunostained tissue sections showed that the number of PMN in the reperfused jejunum was similar to the number of PMN observed in healthy control jejunum samples, as detected by immunohistochemical analysis of HNP1-3 (Fig. 5A. p = 0.90, n = 10). In concordance, analysis of whole jejunum samples for total MPO content by ELISA revealed no increase in MPO protein in the reperfused jejunum, when compared to healthy jejunum control samples (Fig. 5B. I/R = 101.0±31.3 ng/mg vs Healthy = 75.9±32.6 ng/mg. p = 0.71, n = 12). A small number of HNP1-3 positive PMN were observed in cellular debris in the intestinal lumen (Fig. 5A. Picture insert, broad arrow). The presence of these PMN in the intestinal lumen was apparently of no considerable consequence to the remaining jejunum.

### Complement activation and expression in response to IR of the jejunum

**Figure 6:**
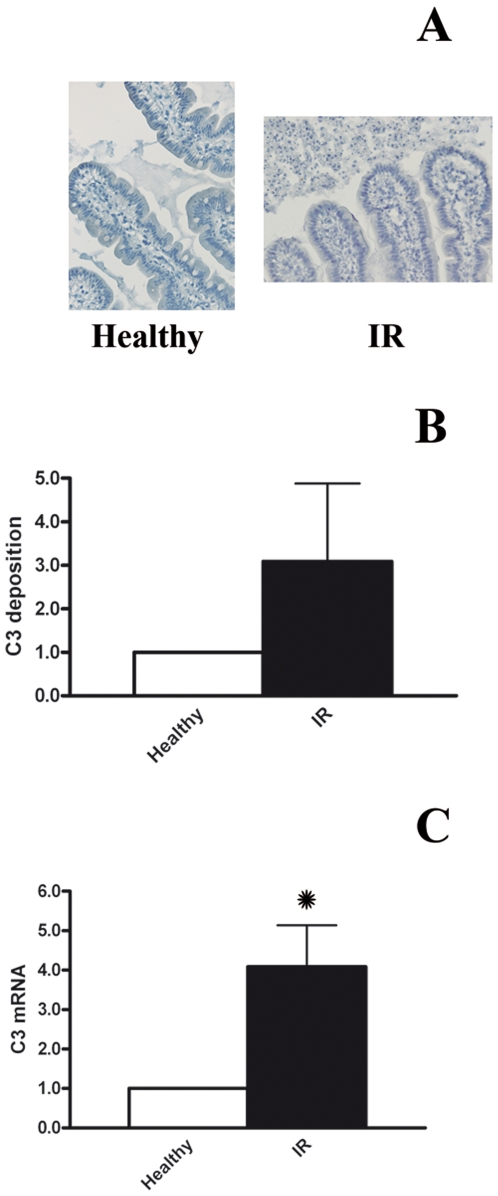
Complement activation. A) C3 was not be detected in the reperfused jejunum using immunohistochemistry. Original magnification 200x. B) Western blot analysis, under reducing conditions, revealed no increase in activated human C3 (±75 kD) in whole reperfused jejunum samples, when compared to healthy control tissue (p = 0.14). C) C3 mRNA expression increased over 4-fold during IR of the human jejunum (p = 0.01).

As a central mediator of IR induced inflammation, complement is involved in the development of organ damage. Complement component C3 is an important constituent of the complement system, of which activation and deposition indicate complement activity. Immunohistochemical analysis of healthy and IR damaged jejunum sections revealed no increase in C3 deposition in response to IR (Fig. 6A). In order to validate these data, human C3 protein in total jejunum tissue after reperfusion was additionally analyzed by Western blot. In keeping with the above results, jejunum tissue specimens showed no increase in the presence of activated C3 over the course of IR, when compared to their healthy controls (Fig. 6B. I/R = 3.09±1.79 relative intensity vs Healthy = 1.00 relative intensity. p = 0.14, n = 12).

Interestingly, subsequent analysis of the response to IR by the jejunum involving C3 mRNA, using Q-PCR analysis, clearly detected C3 mRNA levels in both healthy and IR damaged whole jejunum samples. During reperfusion, the amount of C3 specific mRNA increased over 4 times in response to 30 minutes of ischemia (p = 0.01, Table 2).

## Discussion

The present data demonstrate that rapid shedding of IR damaged intestinal epithelial cells attenuates the development of a classically observed vigorous inflammatory response in the reperfused jejunum. When analyzed over a prolonged reperfusion period, no increase in TNF-α, HO-1, IL-6, IL-8 gene expression, expression of adhesion molecules, PMN influx or activation and deposition of complement were observed. However, HIF1- α and C3 gene expression were up-regulated during reperfusion. This possibly illustrated an IR induced regenerative response, aimed at intestinal barrier restoration and prevention of bacterial translocation.

Development of IR induced organ damage is generally described to be characterized by an excessive and vigorous inflammatory response.[27] This inflammatory response is mainly triggered by apoptosis of cells that cannot be resolved in time by phagocytic cells and will become necrotic in time.[4] Such necrotic cells are a source of damage associated molecular patterns (DAMPS) or alarmins which recruit and activate innate immune cells, aimed at restoration of homeostasis and tissue repair.[28], [29] However, in the context of ischemia and reperfusion such an inflammatory reaction often results in additional tissue damage.

It has been demonstrated in the kidney as well as other organs that IR induced inflammation and subsequent organ damage are dependent on the development of widespread apoptosis.[4] Rapid clearance of apoptotic cells as well as therapeutic strategies to reduce apoptosis have been shown to be critical in preventing the mostly harmful IR induced inflammatory response.[2]–[7], [30]

Conclusive data on intestinal ischemia and reperfusion pathophysiology have been obtained from experimental animal studies. Cytokine activity of TNFα, IL-6 and IL-8 have been associated with the inflammatory reaction following IR.[31] As a prototypic member of the TNF-family, TNF-α is a key mediator of acute inflammation of which expression is readily induced following ischemia reperfusion of the small intestine.[32] Interestingly, we did not detect an increase in TNFα message in our experiments. Similarly, IL-6 was demonstrated as an important component of the acute phase reaction, able to induce tissue injury and inflammation following mesenteric ischemia and reperfusion in IL-6 knockout (KO) mice.[33] IL-6 controls endothelial cell injury, mediating successive neutrophil influx. Similarly, the chemokine IL-8 is involved in intestinal ischemia reperfusion induced inflammation. Administration of an inhibitory anti IL-8 antibody over the course of mesenteric IR in rats prevented neutrophil infiltration and protected the small intestine from IR injury.[34] In keeping with these data, Il-6, IL-8 and TNFα gene expression did not increase in the absence of apoptotic cells, which were shed into the intestinal lumen immediately upon reperfusion.

E-Selectin (CD-62-E and formerly known as endothelial leukocyte adhesion molecule-1 (ELAM-1)) has an important role in the recruitment of leukocytes to sites of tissue injury. Its expression by vascular endothelial cells in response to injury is readily induced by cytokines IL-1 and TNFα. Interestingly, our data suggest that despite initial presence of damaged epithelial cells in response to an IR period, the rapid shedding of damaged epithelial debris largely averts endothelial cell activation. Consistent with this observation, PMN infiltration, assessed by total MPO tissue levels or the presence of HNP1-3 positive cells, in IR was not detected in our experiments. MPO, most abundantly released upon PMN activation, has been demonstrated to have a clear effect on the development of IR induced organ damage. Renal IR studies have demonstrated that MPO itself is able to contribute to the development of organ damage.[35]

As a central innate immune regulator, the complement system is activated during intestinal IR and contributes substantially to IR induced inflammation, organ damage and failure.[8], [9], [36] Preventing complement activation has been proven to be beneficial to organ function after IR in general and intestinal IR more specifically.[8], [12], [20] Central in the activation of complement is the formation of C3a, an anaphylatoxin with the ability to attract inflammatory cells into the reperfused ischemic tissue. In our model no deposition of activated C3 in reperfused jejunum was detected, using immunohistochemical and Western blot analysis. Interestingly, our results do however suggest C3 gene expression in healthy jejunum tissue as well as an increased synthesis of C3 in response to IR. The lack of activated complement components as well as a clear increase in local C3 mRNA expression levels may illustrate the intestines response aimed at protection and preservation. The ability of small intestinal epithelial cells to express biological mediators such as C3 in response to IR was to be expected since previous work demonstrated C3 gene expression in inflamed small intestine.[37] To the best of our knowledge, however, these are the first results that demonstrate C3 mRNA expression in the healthy jejunum. Further analysis will have to elucidate whether increased expression of immune regulatory proteins is directed at preventing gut barrier bacterial translocation. However, when faced with massive intestinal barrier failure, increased expression of immune regulatory proteins able to prevent gut barrier bacterial translocation might be exceedingly useful.

The recently discovered molecular mechanism that recognizes and responds to excessive cell death consisting of SAP130 and the macrophage inducible C-type lectin (Mincle) fits in our observations.[38] The loss of excessive numbers of dead cells into the gut lumen prevents SAP130 released by the dead cells to reach tissue macrophages thus largely preventing the production of inflammatory cytokines driving rapid neutrophil infiltration. Similarly activation of complement by dead cells is prevented and maybe therefore the PMN chemotactic factors C3a en C5a will not be produced.

A mechanism of protection was suggested by the enhanced expression of HIF-1α in the surviving epithelium. Expression of HIF-1α in response to low oxygen tension during ischemia and early reperfusion triggers physiologic responses characterized by activation of functional proteins mucin, P-glycoprotein, intestinal trefoil factor and adenosine A2B receptor, aimed at preventing mucosal inflammation.[39] The role in preserving gut wall integrity in response to IR as well as the exciting role of HIF-1α in different cardiac and brain pre-conditioning IR models underlines a possibly protective influence of increased HIF-1α mRNA expression as detected in our model in response to IR of the human intestine.[39]–[42]

Taken together, ischemia, sensed by the small intestine, induces normal physiological and IR induced responses. Our data provide new and compelling evidence of increased HIF-1α and also C3 gene expression during the reperfusion period. However, these responses are not paralleled by an IR induced inflammatory response, since important conditions to an inflammatory reaction have not been met by the absence of dead cells in the reperfused tissue. It is important to realize that these data indicate that the human intestine is more resistant to IR than initially thought. Its ability to shed damaged epithelial cells and repair its ever important barrier function without triggering massive inflammation can be seen as key features that prevent the gut from inflammation following splanchnic ischemia and reperfusion.

## References

[1] Derikx JPM, Matthijsen RA, de Bruïne AP, van Bijnen AA, Heineman E (2008). Rapid Reversal of Human Intestinal Ischemia-Reperfusion Induced Damage by Shedding of Injured Enterocytes and Reepithelialisation.. PLoS ONE.

[2] Yellon DM, Hausenloy DJ (2007). Myocardial reperfusion injury.. N Engl J Med.

[3] de Groot H, Rauen U (2007). Ischemia-reperfusion injury: processes in pathogenetic networks: a review.. Transplant Proc.

[4] Daemen MA, van 't Veer C, Denecker G, Heemskerk VH, Wolfs TG (1999). Inhibition of apoptosis induced by ischemia-reperfusion prevents inflammation.. J Clin Invest.

[5] Donath S, Li P, Willenbockel C, Al-Saadi N, Gross V (2006). Apoptosis repressor with caspase recruitment domain is required for cardioprotection in response to biomechanical and ischemic stress.. Circulation.

[6] Yaoita H, Ogawa K, Maehara K, Maruyama Y (1998). Attenuation of ischemia/reperfusion injury in rats by a caspase inhibitor.. Circulation.

[7] Restifo NP (2000). Building better vaccines: how apoptotic cell death can induce inflammation and activate innate and adaptive immunity.. Curr Opin Immunol.

[8] Hart ML, Ceonzo KA, Shaffer LA, Takahashi K, Rother RP (2005). Gastrointestinal ischemia-reperfusion injury is lectin complement pathway dependent without involving C1q.. J Immunol.

[9] Zhou W, Farrar CA, Abe K, Pratt JR, Marsh JE (2000). Predominant role for C5b-9 in renal ischemia/reperfusion injury.. J Clin Invest.

[10] Romson JL, Hook BG, Kunkel SL, Abrams GD, Schork MA (1983). Reduction of the extent of ischemic myocardial injury by neutrophil depletion in the dog.. Circulation.

[11] Libby P, Maroko PR, Bloor CM, Sobel BE, Braunwald E (1973). Reduction of experimental myocardial infarct size by corticosteroid administration.. J Clin Invest.

[12] Wada K, Montalto MC, Stahl GL (2001). Inhibition of complement C5 reduces local and remote organ injury after intestinal ischemia/reperfusion in the rat.. Gastroenterology.

[13] Litt MR, Jeremy RW, Weisman HF, Winkelstein JA, Becker LC (1989). Neutrophil depletion limited to reperfusion reduces myocardial infarct size after 90 minutes of ischemia. Evidence for neutrophil-mediated reperfusion injury.. Circulation.

[14] Daemen MA, van de Ven MW, Heineman E, Buurman WA (1999). Involvement of endogenous interleukin-10 and tumor necrosis factor-alpha in renal ischemia-reperfusion injury.. Transplantation.

[15] Frangogiannis NG, Smith CW, Entman ML (2002). The inflammatory response in myocardial infarction.. Cardiovasc Res.

[16] Takada M, Nadeau KC, Shaw GD, Marquette KA, Tilney NL (1997). The cytokine-adhesion molecule cascade in ischemia/reperfusion injury of the rat kidney. Inhibition by a soluble P-selectin ligand.. J Clin Invest.

[17] Lucchesi BR (1990). Modulation of leukocyte-mediated myocardial reperfusion injury.. Annu Rev Physiol.

[18] de Vries B, Kohl J, Leclercq WK, Wolfs TG, van Bijnen AA (2003). Complement factor C5a mediates renal ischemia-reperfusion injury independent from neutrophils.. J Immunol.

[19] Guo RF, Ward PA (2005). Role of C5a in inflammatory responses.. Annu Rev Immunol.

[20] De Vries B, Matthijsen RA, Wolfs TG, Van Bijnen AA, Heeringa P (2003). Inhibition of complement factor C5 protects against renal ischemia-reperfusion injury: inhibition of late apoptosis and inflammation.. Transplantation.

[21] Murohara T, Delyani JA, Albelda SM, Lefer AM (1996). Blockade of platelet endothelial cell adhesion molecule-1 protects against myocardial ischemia and reperfusion injury in cats.. J Immunol.

[22] Schoenberg MH, Poch B, Younes M, Schwarz A, Baczako K (1991). Involvement of neutrophils in postischaemic damage to the small intestine.. Gut.

[23] Ma XL, Tsao PS, Lefer AM (1991). Antibody to CD-18 exerts endothelial and cardiac protective effects in myocardial ischemia and reperfusion.. J Clin Invest.

[24] Matthijsen RA, Derikx JP, Steffensen R, van Dam RM, Dejong CH (2009). Mannose-binding lectin null alleles are associated with preserved epithelial cell integrity following intestinal ischemia reperfusion in man.. Mol Immunol.

[25] Kanda T, Fujii H, Tani T, Murakami H, Suda T (1996). Intestinal fatty acid-binding protein is a useful diagnostic marker for mesenteric infarction in humans.. Gastroenterology.

[26] Engelberts I, Stephens S, Francot GJ, van der Linden CJ, Buurman WA (1991). Evidence for different effects of soluble TNF-receptors on various TNF measurements in human biological fluids.. Lancet.

[27] Vedder NB, Winn RK, Rice CL, Chi EY, Arfors KE (1990). Inhibition of leukocyte adherence by anti-CD18 monoclonal antibody attenuates reperfusion injury in the rabbit ear.. Proc Natl Acad Sci U S A.

[28] Bianchi ME (2007). DAMPs, PAMPs and alarmins: all we need to know about danger.. J Leukoc Biol.

[29] Lotze MT, Zeh HJ, Rubartelli A, Sparvero LJ, Amoscato AA (2007). The grateful dead: damage-associated molecular pattern molecules and reduction/oxidation regulate immunity.. Immunol Rev.

[30] Huynh ML, Fadok VA, Henson PM (2002). Phosphatidylserine-dependent ingestion of apoptotic cells promotes TGF-beta1 secretion and the resolution of inflammation.. J Clin Invest.

[31] Grotz MR, Deitch EA, Ding J, Xu D, Huang Q (1999). Intestinal cytokine response after gut ischemia: role of gut barrier failure.. Ann Surg.

[32] Salvemini D, Wang ZQ, Zweier JL, Samouilov A, Macarthur H (1999). A nonpeptidyl mimic of superoxide dismutase with therapeutic activity in rats.. Science.

[33] Cuzzocrea S, De Sarro G, Costantino G, Ciliberto G, Mazzon E (1999). IL-6 knock-out mice exhibit resistance to splanchnic artery occlusion shock.. J Leukoc Biol.

[34] Tsuruma T, Yagihashi A, Tarumi K, Hirata K (1998). Anti-rat IL-8 (CINC) monoclonal antibody administration reduces ischemia-reperfusion injury in small intestine.. Transplant Proc.

[35] Matthijsen RA, Huugen D, Hoebers NT, de Vries B, Peutz-Kootstra CJ (2007). Myeloperoxidase is critically involved in the induction of organ damage after renal ischemia reperfusion.. Am J Pathol.

[36] Arumugam TV, Shiels IA, Woodruff TM, Granger DN, Taylor SM (2004). The role of the complement system in ischemia-reperfusion injury.. Shock.

[37] Laufer J, Oren R, Goldberg I, Horwitz A, Kopolovic J (2000). Cellular localization of complement C3 and C4 transcripts in intestinal specimens from patients with Crohn's disease.. Clin Exp Immunol.

[38] Yamasaki S, Ishikawa E, Sakuma M, Hara H, Ogata K (2008). Mincle is an ITAM-coupled activating receptor that senses damaged cells.. Nat Immunol.

[39] Taylor CT, Colgan SP (2007). Hypoxia and gastrointestinal disease.. J Mol Med.

[40] Bergeron M, Gidday JM, Yu AY, Semenza GL, Ferriero DM (2000). Role of hypoxia-inducible factor-1 in hypoxia-induced ischemic tolerance in neonatal rat brain.. Ann Neurol.

[41] Cai Z, Manalo DJ, Wei G, Rodriguez ER, Fox-Talbot K (2003). Hearts from rodents exposed to intermittent hypoxia or erythropoietin are protected against ischemia-reperfusion injury.. Circulation.

[42] Patel A, van de Poll MC, Greve JW, Buurman WA, Fearon KC (2004). Early stress protein gene expression in a human model of ischemic preconditioning.. Transplantation.

